# Pathological and functional significance of Semaphorin-5A in pancreatic cancer progression and metastasis

**DOI:** 10.18632/oncotarget.23644

**Published:** 2017-12-23

**Authors:** Sugandha Saxena, Yuri Hayashi, Lingyun Wu, Mohammad Awaji, Pranita Atri, Michelle L. Varney, Abhilasha Purohit, Satyanarayana Rachagani, Surinder K. Batra, Rakesh K. Singh

**Affiliations:** ^1^ Department of Pathology and Microbiology, University of Nebraska Medical Center, Omaha, NE 68198, USA; ^2^ Department of Biochemistry and Molecular Biology, University of Nebraska Medical Center, Omaha, NE 68198, USA; ^3^ Department of Internal Medicine, University of Nebraska Medical Center, Omaha, NE 68198, USA; ^4^ The Wistar Institute of Anatomy and Biology, Philadelphia, PA 19104, USA

**Keywords:** Semaphorin-5A, pancreatic cancer, metastasis, pancreatic neuroendocrine tumors, Plexin-B3

## Abstract

Semaphorin-5A (SEMA5A) has differential cell surface expression between normal and cancer cells and represents an attractive target for therapeutic intervention in pancreatic cancer (PC). In this study, we delineated the pathological expression and significance of SEMA5A during PC progression and metastasis. We utilized human tissue microarrays and different PC mouse models (Pdx1-cre; LSL- Kras^(G12D)^, Pdx1-Cre; LSL-Kras^(G12D)^; LSL-p53^(R172H)^ and RIP1-Tag2) to analyze SEMA5A expression during PC progression. Using human patients and different mouse models, we demonstrated that SEMA5A expression was highest in liver metastases, followed by primary pancreatic tumors, and the lowest expression was found in the normal pancreas. SEMA5A expression was localized on tumor cells with no staining in the surrounding stroma. To understand the functional significance of SEMA5A, we treated PC cell lines with recombinant SEMA5A. We observed an increase in migration, chemotaxis, and scattering of PC cells. To delineate the signaling axis of SEMA5A, we generated SEMA5A receptor-Plexin-B3 knockdown in T3M-4 and CD18/HPAF PC cell lines and observed that the effect of SEMA5A treatment was absent in the Plexin-B3 knockdown counterparts of T3M-4 and CD18/HPAF cells. SEMA5A treatment leads to phosphorylation of cMET in Plexin-B3 dependent manner. Our data demonstrate that there is an increase in SEMA5A expression during PC progression and the elevation of this expression takes place at metastatic sites especially the liver in both exocrine and endocrine tumors. SEMA5A can elicit a migratory response in cells by activating cMET through the Plexin-B3 receptor. In conclusion, SEMA5A signaling represents a potential molecule for targeting metastasis in pancreatic cancer.

## INTRODUCTION

Semaphorins are well known for their function as guidance and migration cue molecules designed to help cell reach the target site during embryonic development of the nervous system and cardiovascular patterning [1–3]. Recent studies have shown that aberrant expression of axonal guidance cue molecules such as semaphorins in cancer, and their dysregulated signaling plays a significant role in cancer angiogenesis and metastasis [4–9]. Several studies have demonstrated the expression and role of subclass-5 member Semaphorin-5A (SEMA5A) in various malignancies including glioma, lung, prostate, pancreas, melanoma and gastric [10–23]. SEMA5A has not been associated with a univocal role in cancer [17], as it acts as a tumor promoter in cancers like gastric [14–16], prostate [20] and, ovary [23] while it acts as a tumor suppressor in glioma [11, 12] and lung cancer [13]. This contradictory functions of this molecule can be attributed to the possible interaction of SEMA5A with different receptors and thereby activation of divergent pathways in a tissue-specific manner [17]. The variable response of SEMA5A towards differences in extracellular matrix components of different tissues [24] can also be a potential reason as the molecule itself has bifunctionality due to presence of two opposing domains (Sema and Thrombospondin).

Our group identified SEMA5A as a putative cell adhesion molecule involved in organ-specific homing of PC cells [20] and plays an significant role PC angiogenesis [18] and metastasis [19, 22]. We demonstrated that the overexpression of both membrane-bound and secretory SEMA5A in a PC cells resulted in enhanced metastasis in PC cell line derived xenograft mouse model [19, 22] . Thus, to gain a clearer understanding of the pathological and functional role of SEMA5A in PC, we evaluated the expression and function of SEMA5A using different disease progression models and *in vitro* functional assays. In the present study, we report an increase in SEMA5A expression during PC progression and at different metastatic sites. This increase in SEMA5A expression induces cellular migration in PC cells by activating cMET through Plexin-B3 receptor. To summarize, the SEMA5A axis represents a potential target that can be exploited for the development of future PC diagnosis and therapies.

## RESULTS

### SEMA5A expression increases in pancreatic tumors with disease progression and depends on the differentiation status

**Figure 1:**
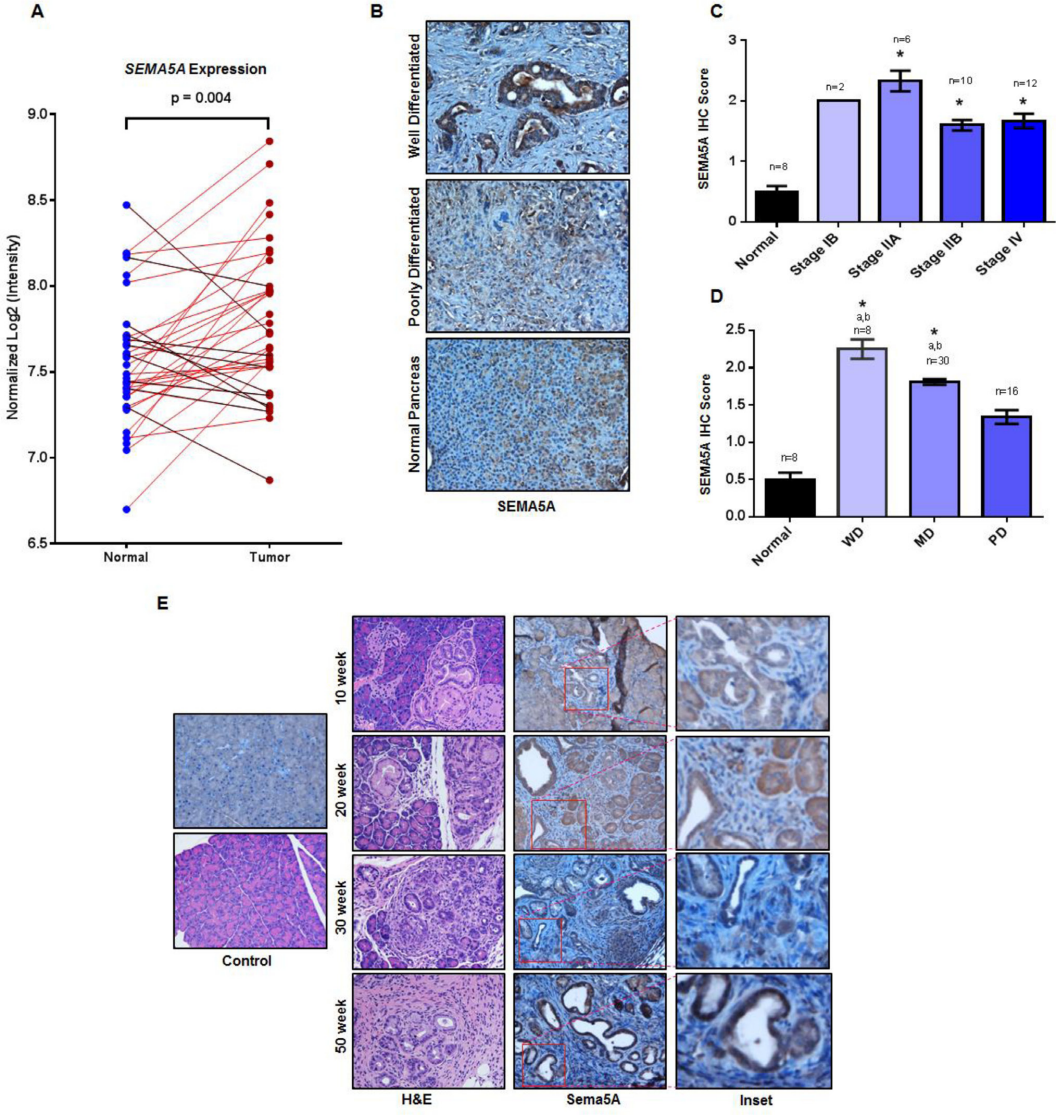
Increase in SEMA5A expression with PC disease progression (**A**) *SEMA5A* gene expression analysis in the paired pancreatic tumor and normal tissue controls present in the GEO PDAC microarray dataset GDS4103 containing datasets of 39 patients. The paired analysis shows significantly higher expression (*p* = 0.004) of SEMA5A in tumor samples in comparison with their corresponding normal control samples. (**B**) Representative images showing SEMA5A expression in normal pancreas, primary well differentiated and poorly differentiated PC in human patients (200×). (**C**, **D**) Bar graph showing SEMA5A IHC score between tumors at different cellular differentiation status and normal tissues and different stages of tumors and normal tissues. Shown with symbol “a” (^a^) are the significant statistical differences (*p* ≤ 0.05) between various tumors and the normal controls. Similarly, the symbol “b” (^b^) represents significant statistical differences (*p* ≤ 0.05) between various tumors and poorly/undifferentiated tumors. SEMA5A expression in tumor tissue and normal pancreas in TMA was examined using IHC. The values are mean IHC composite score ± Standard Error of Mean (SEM). The significance of the data was determined by the non-parametric Mann–Whitney *U*-test (two-tailed). A value of *p* < 0.05 was deemed significant. (**E**) Representative images of Sema5A immunohistochemistry performed on tumors from KC mice at different ages (10 week, 20 week, 30 week and 50 week) demonstrating progressively increasing Sema5A expression. The normal pancreas of 50 week Pdx1-cre mice is negative for Sema5A expression (200×).

We utilized the publicly available GEO dataset, GDS4103, to analyze the expression of *SEMA5A* in PC. This dataset contains 39 pancreatic ductal adenocarcinoma tumors, and corresponding matched normal samples. First, we analyzed the distribution of *SEMA5A* expression values for both tumor samples and matched controls using box plot analysis to find outliers in the data. (Supplementary Figure 1). We observed four outliers in the distribution of *SEMA5A* expression values of normal samples. We removed these outliers for our analysis, performed a paired Student *t*-test to analyze this data, and observed significantly higher expression (*p* = 0.004) of *SEMA5A* in tumor samples in comparison with their matched controls (Figure 1A).

Next, we performed SEMA5A immunohistochemistry (IHC) analysis on a tissue microarray (TMA) containing 33 cases of human patients with PC and eight unmatched normal pancreatic tissues. SEMA5A expression was observed mainly in PC tumors whereas normal tissue showed either low or no expression (Figure 1B). Moreover, localization of SEMA5A expression was on the membrane of tumor cells with no positive staining in surrounding stroma (Figure 1B). There was a significant difference (*p* < 0.05) in SEMA5A expression between normal pancreatic tissues and different stages of PC. Furthermore, analysis of SEMA5A expression within different stages of PC revealed a lower SEMA5A expression for advanced stages like Stage IIB and Stage IV than initial stages like Stage IB or IIA, but this difference in SEMA5A expression was not significant (Figure 1C). We analyzed SEMA5A expression on differentiation status of the tumor and observed that SEMA5A expression was significantly higher (*p* < 0.05) in well differentiated and moderately differentiated tumors in comparison with poorly/undifferentiated tumors as well as normal pancreatic tumors (Figure 1D).

Since recent literature suggests aberrant genomic expression of axonal guidance cue molecules in cancer [25], we went ahead and analyzed the copy number variation of *SEMA5A* in PC cases using The Cancer Genome Atlas (TCGA) Database. We found gain of *SEMA5A* gene in 35 out of 49 PC cases (Supplementary Figure 2). The gain of *SEMA5A* gene ranged between the value of 2 to 3 of copy number in a diploid cell. However, aberrant gene expressions have a gain of copy number greater than 3.

To gain an insight of Sema5A expression during PC disease progression, we utilized mouse PC tissues collected at different stages (10, 20, 30 and 50 weeks) of PC progression in Pdx1-cre; LSL- Kras^(G12D)^ (KC) mouse model. We observed expression of Sema5A is absent in the normal pancreas, derived from the control Pdx1-Cre mice. However, in KC mice, we observed Sema5A expression as early as 10-weeks of age (Figure 1E). Its expression progressively increased as disease advanced from 20, 30 and 50 weeks of PC progression. Similar to SEMA5A expression in human PC tissues, localization of Sema5A expression was observed on membrane of tumor cells with no staining in surrounding stroma (Figure 1E). In addition, we observed non-specific background staining in acinar comportment of the pancreas.

### SEMA5A expression is higher at metastatic sites

**Figure 2:**
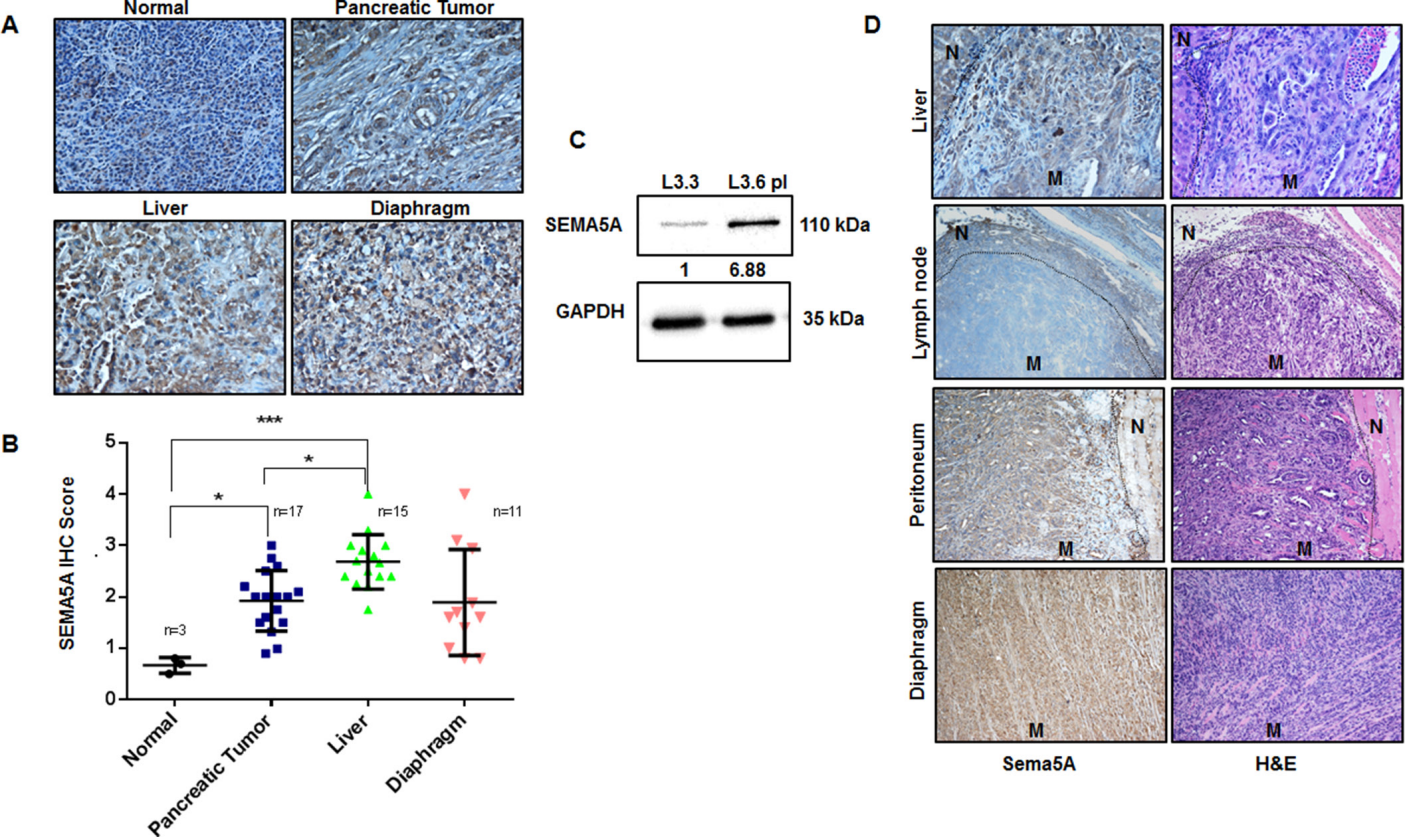
Expression of SEMA5A progressively increases at metastatic sites of PC (**A**) Representative images showing SEMA5A expression in normal pancreas, primary PC and metastatic sites including liver and diaphragm in human patients (200×). (**B**) The graph shows higher SEMA5A IHC Score at metastatic sites in comparison with the primary tumor, evaluated using TMA with 21 cases of PC patients. The values are mean IHC composite score ± SEM. We found the highest IHC scoring of SEMA5A at liver metastatic site (*n* = 15) with a significant (*p* ≤ 0.001) difference between normal (*n* = 3) and primary cancer (*n* = 17). The significance of the data was determined using one-way ANOVA with (^*^p ≤ 0.05, ^**^p ≤ 0.01, ^***^p ≤ 0.001, ^ns^*p* > 0.05). (**C**) Western blot analysis showing higher SEMA5A expression in L3.6pl (high metastatic potential) in comparison with L3.3 (low metastatic potential) cell lines using GAPDH as a loading control. Quantification of the intensity of the SEMA5A bands with respect to the loading control GAPDH was performed by Image J software. Bands were normalized to the L3.3 cells. (**D**) Representative images of Sema5A immunohistochemistry performed on metastatic sites (mesenchymal lymph node (*n* = 1), liver (*n* = 5), diaphragm (*n* = 1), and peritoneum (*n* = 2) of KPC mice, demonstrating that metastatic sites are positive for the expression of Sema5A except lymph node (200×). “N” represents normal tissue while “M” represents metastasis.

With an interest to evaluate SEMA5A expression between primary cancer and different metastatic sites, we utilized TMA with 21 cases of PC patients with primary tumor and either one, two or three metastatic sites like liver, diaphragm, and intestine. IHC scoring of SEMA5A revealed significantly higher expression of SEMA5A in liver metastatic site compared to normal pancreas and primary PC tissue (Figure 2A, 2B). There was no difference in SEMA5A expression at diaphragmatic metastatic lesions and the primary tumor of human PC (Figure 2A, 2B). Our SEMA5A antibody showed non-specific background staining of SEMA5A for normal liver cells. To gain insight into functionality of SEMA5A we also used this TMA to derive correlational analysis of SEMA5A expression and angiogenesis marker CD31 and CXCL8, and apoptosis marker CC3. We found very weak but significant correlation of SEMA5A with CXCL8 (R^2^ = 0.158, *p* = 0.004) and Cleaved Caspase-3 (R^2^ = 0.125, *p* = 0.009) and no significant correlation with CD31 (R^2^ = 0.014, *p* = 0.4).

We also analyzed SEMA5A expression in L3.3 and L3.6pl cell lines; variants with different metastatic potential derived from fast-growing FG/COLO-357 cells as described by Bruns *et al.* (1999) in their *in vivo* selection study using orthotopic implantation in nude mice [26]. We observed higher SEMA5A expression in highly metastatic L3.6pl cells derived from liver metastasis than the low metastatic potential L3.3 cells derived from primary tumor (Figure 2C).

To further strengthen our observation, we evaluated Sema5A expression at different metastatic sites from Pdx1-Cre; LSL-Kras^(G12D)^; LSL-p53^(R172H)^ (KPC) mice. We examined tissues from different metastatic sites including liver, spleen, lymph node, peritoneum, and diaphragm from KPC mice sacrificed at the 50-week time point. We observed that Sema5A expression was positive for all the metastatic sites except the lymph nodes (Figure 2D).

### *Sema5A* expression is high at angiogenic islets and metastasis like primary (MLP) tumors in PanNET

**Figure 3:**
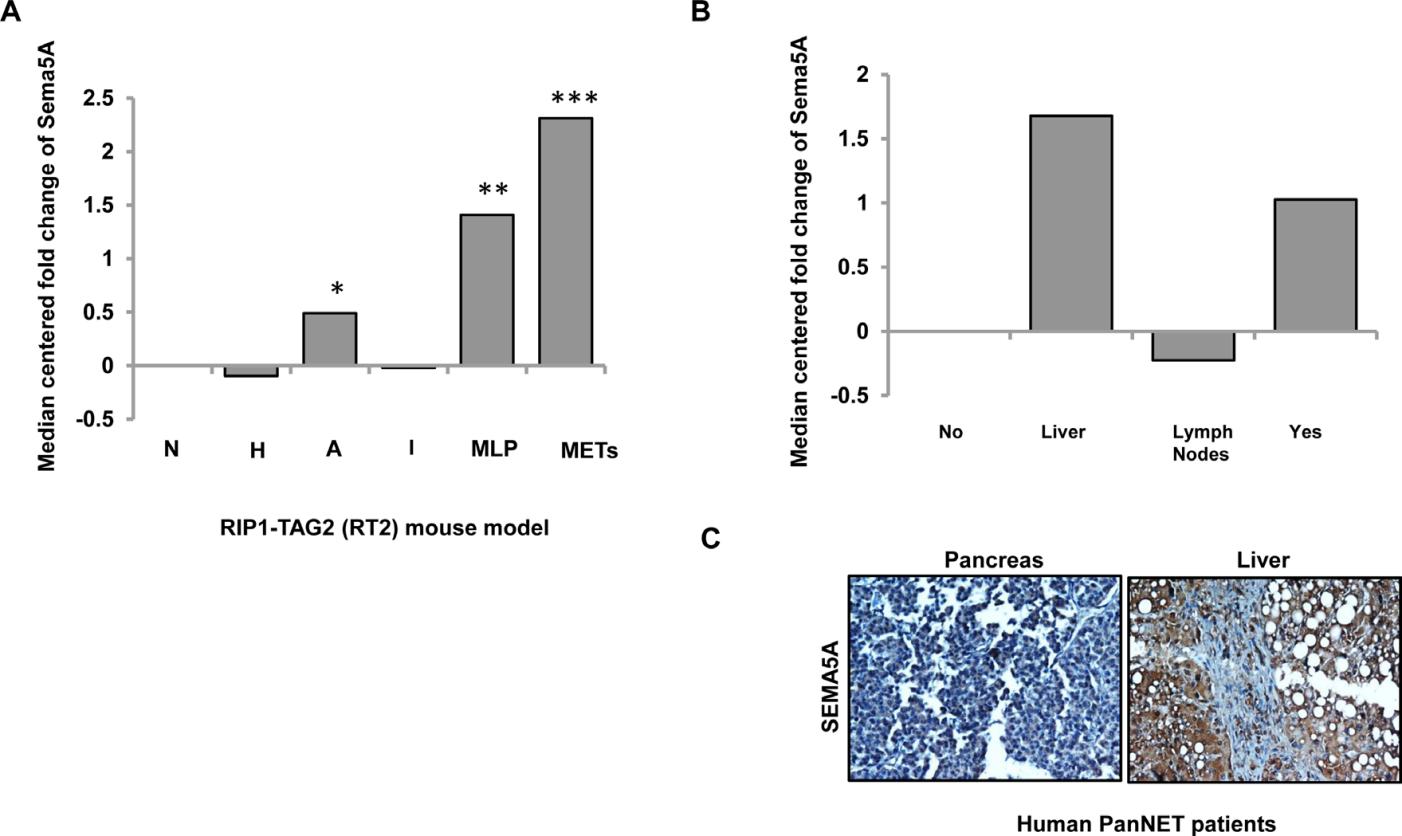
Sema5A expression is higher in metastasis in comparison with normal islets in PanNET (**A**) Expression of *Sema5A* as median-centered fold change with respect to normal islets (N; *n* = 3) in different stages (hyperplastic/dysplastic (H; *n* = 3) islets and angiogenic (A; *n* = 3) islets), tumor subtypes (*n* = 10; classified into Insulinoma/Islet Tumors (IT) and Metastasis Like Primary (MLP) Tumors) and liver metastases (Mets; *n* = 3) of RT2 mouse model of PanNET. Angiogenic islets, MLP tumors, and liver metastases show upregulation of Sema5A with respect to normal islets. (**B**) Expression of *SEMA5A* as median-centered fold change with respect to normal islets in primary tumor with (Yes) or without (No) metastasis and liver (Liver) and lymph node (Lymph Node) metastases from human PanNETs. This dataset included four normal islets, 75 primary tumors positive or negative for metastasis and seven samples of either liver or lymph node metastases. The significance of the data is determined using Student *t*-test (^*^p ≤ 0.05, ^**^p ≤ 0.01, ^***^p ≤ 0.001, ^ns^*p* > 0.05). (**C**) Representative images of SEMA5A IHC performed on pancreas and liver of a patient suffering from PanNET (200×).

We investigated the *Sema5A* mRNA expression profiles from the progressive stages of the disease in RT2 mice. Sadanandam *et al.* (2015) in their study of cross-species analysis of PanNET provides a description of parameters for hierarchical clustering and resulting classified groups [27]. The different stages of disease progression described in this paper are normal (N), hyperplastic (H), and angiogenic (A) islets. Furthermore, classification of tumor samples by their similarities with the metastasis resulted in the formation of two distinct groups called MLP and well-differentiated islet/insulinoma tumors (IT). As the name suggests, gene expression of MLP was similar to the gene expression at metastatic sites in the RT2 model. This dataset included pools of normal (*n* = 3), hyperplastic/dysplastic (*n* = 3), and angiogenic (*n* = 3) islets, as well as individual tumors (*n* = 10) and liver macrometastases (Mets; *n* = 3). In this mRNA evaluation, we found upregulation of *Sema5A* expression in liver metastasis in comparison with normal islets (Figure 3A) followed by MLP and angiogenic islets. On the other hand, hyperplastic/dysplastic islets and well differentiated IT showed downregulation of *Sema5A* expression in comparison with normal islets.

Next, *SEMA5A* expression was evaluated in human mRNA transcriptomes of PanNET patients using NMF consensus clustering analysis as described by Sadanandam *et al.* in a cross-species analysis of PanNET [27]. This mRNA transcriptome had core clinical gene expression information of 86 patients classified as nonfunctional PanNETs, insulinomas, and normal pancreatic islet samples [28]. This dataset included four normal islets, 75 primary tumors positive or negative for metastasis and seven samples of either liver or lymph node metastases. The highest *SEMA5A* mRNA expression was found in liver metastasis in comparison with normal islets (Figure 3B) followed by primary tumors positive for metastasis. In contrast, *SEMA5A* mRNA expression was down-regulated in lymph node metastasis, and we observed no change of *SEMA5A* expression in patients negative for metastasis. In support of this observation, we also found higher *SEMA5A* in liver metastasis in comparison with the corresponding primary tumor (Figure 3C) in the tissue core of a single case of PanNET patient present in TMA with cases of PC patients with primary tumor and metastatic sites as described above.

### SEMA5A enhanced cellular migration of PC cells

**Figure 4:**
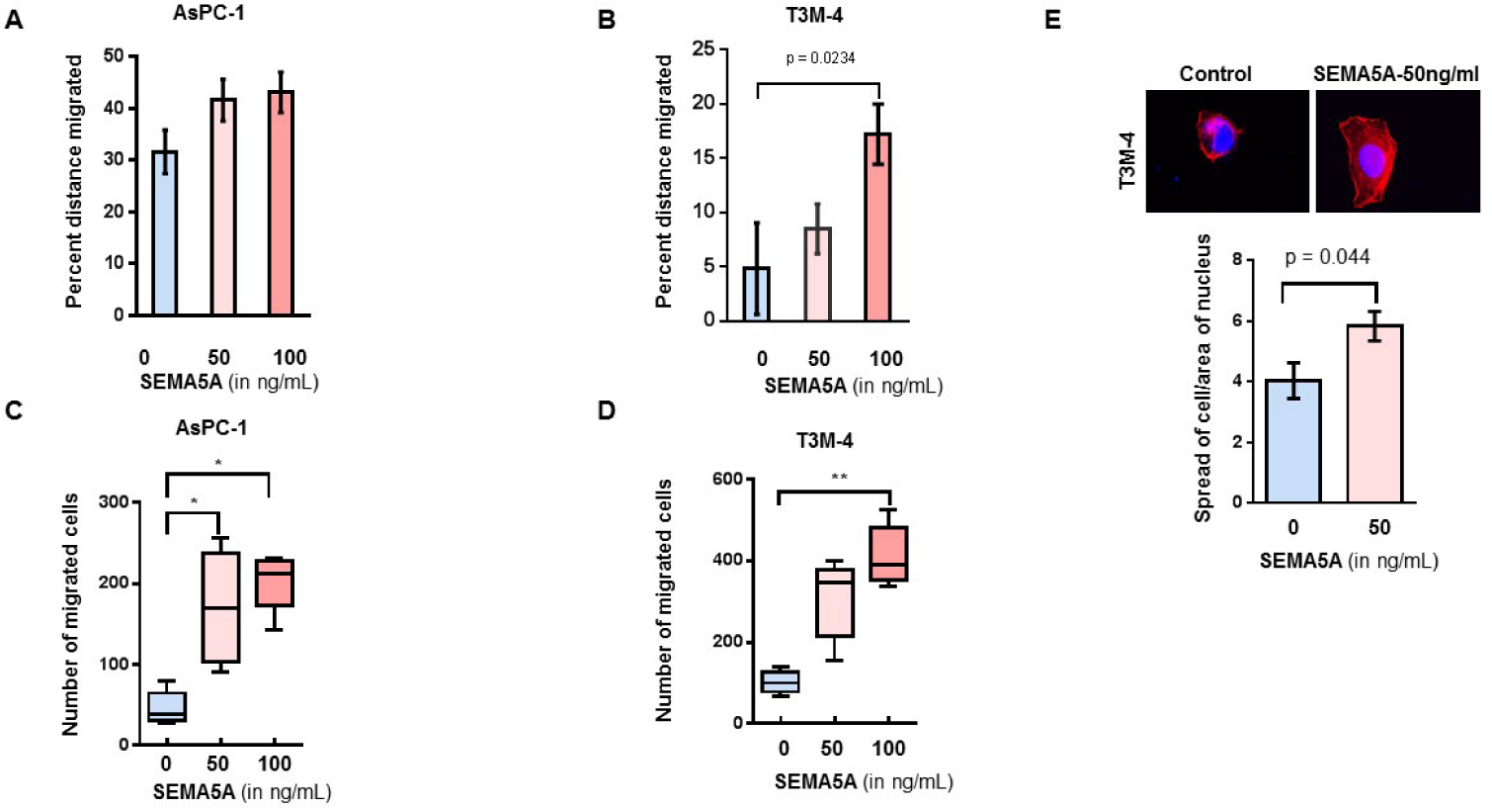
SEMA5A treatment induced cellular migration of PC cells (**A**, **B**) Bar graph showing a higher percent distance migrated in AsPC-1 (A) and T3M-4 (B) cell lines treated with 50 ng/mL or 100 ng/mL of SEMA5A in comparison with untreated cells. Wound scratch assay was used to evaluate migration. The values are a mean percentage of migration ± SEM. (**C**, **D**) Box plot graphs showing chemotaxis of AsPC-1 (C) and T3M-4 (D) towards SEMA5A. Using a transwell migration assay, chemotaxis was evaluated in response to different SEMA5A concentration (0, 50 ng or 100 ng)/mL. The migrated cells were counted in five independent fields. The values are a mean number of migrated cells ± SEM. The significance of the data was determined using the Kruskal–Wallis test by ranks (^*^p ≤ 0.05, ^**^p ≤ 0.01, ^***^p ≤ 0.001, ^ns^*p* > 0.05). (**E**) Representative images of cellular spread in the presence or absence of SEMA5A (50ng/mL) (400×). Bar graph showing an increase (*p* = 0.04) in the ratio of the area of cellular spreading with respect to the area of the nucleus of T3M-4 cells treated with 50 ng/mLof SEMA5A for 30 min. Area of cellular spread and area of their respective nucleus was calculated for 15 different SEMA5A-treated and -untreated cells using Image J Software. The values are mean of the ratio of cellular area and area of nucleus ± SEM. The significance of the data was determined using the Mann–Whitney *U*-Test.

To evaluate the effect of SEMA5A on the migration of PC cells, we treated AsPC-1 and T3M-4 cells with two different concentrations (50 ng/mL and 100 ng/mL) of recombinant SEMA5A. We observed a higher percentage of wound closure/migration in both AsPC-1 (Figure 4A) and T3M-4 (Figure 4B) cells with recombinant human SEMA5A treatment in comparison with their respective untreated control cells. We found a significant difference in the wound closure ability of T3M-4 cells treated with 100 ng/mL of SEMA5A in comparison with untreated cells (*p* = 0.0234). Similarly, we observed chemotaxis of both AsPC-1 (Figure 4C) and T3M-4 (Figure 4D) towards SEMA5A. A higher number of AsPC-1 cells migrated towards 50 ng/mL and 100 ng/mL in comparison with the untreated control cells. There was a significant difference in the number of T3M-4 cells that had migrated towards 100 ng/mL (*p* < 0.001) in comparison with the untreated control cells. We observed an increase (*p* = 0.04) in cellular protrusion or cellular spreading of T3M-4 cells when treated with 50 ng/mL of SEMA5A for 30 min (Figure 4E). We also evaluated the effect of SEMA5A on cellular proliferation by MTT assay using PC cell lines AsPC-1, T3M-4 and CD18/HPAF and observed that there is no differences in cellular proliferation of these cell lines upon SEMA5A treatment (Supplementary Figure 3A–3E).

### SEMA5A enhances PC cell migration by activating cMet signaling in a Plexin-B3-dependent manner

**Figure 5:**
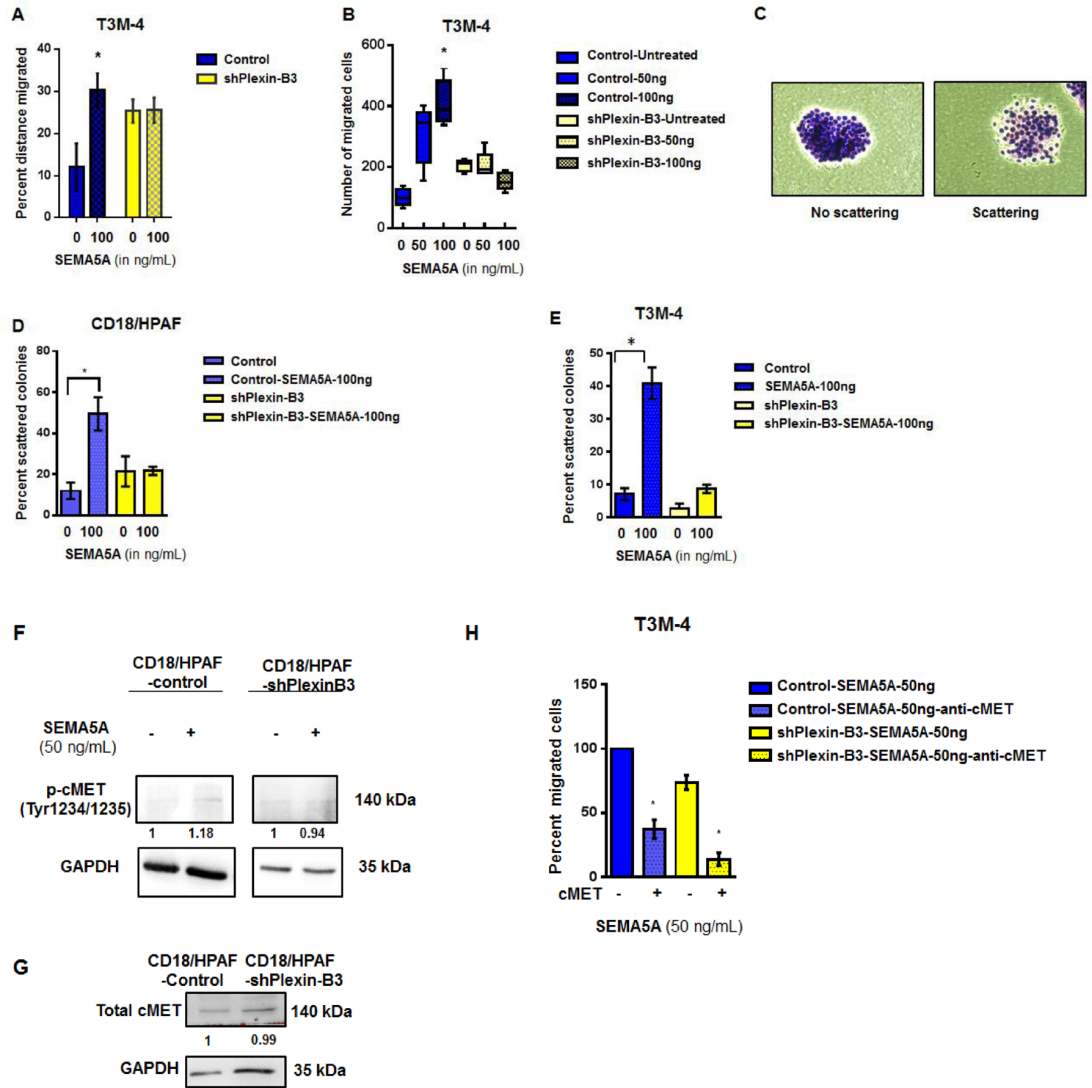
SEMA5A activates cMET signaling and results in increased migration in a Plexin-B3 dependent manner (**A**) Bar graph showing the percent distance migrated of T3M-4 Control and Plexin*-*B3 knockdown cells with and without SEMA5A treatment (50 ng/mL). Wound healing assay was used for evaluation of migration of the T3M-4 control and knockdown cells. The values are a mean percentage of migration ± SEM. The significance of the data was calculated using Two-way ANOVA test. (**B**) Box plot graph showing the chemotactic ability of Control and Plexin-B3 knockdown cells towards SEMA5A. The migrated cells were counted in five independent fields (100×), using a Nikon Eclipse E800 microscope The values are a mean number of migrated cells ± SEM. The significance of the data was determined using Kruskal–Wallis test by ranks (^*^p ≤ 0.05, ^**^p ≤ 0.01, ^***^p ≤ 0.001, ^ns^*p* > 0.05). (**C**) Representative images of nonscattered and scattered colonies (200×). (**D**, **E**) Bar graph of scattering potential of Control and Plexin-B3 knockdown cells towards SEMA5A. The graph shows a higher scattering of colonies in both CD18/HPAF (D) and T3M-4 (E), Control cells with SEMA5A treatment in comparison to their respective untreated control cells. The percent of scattered colonies was calculated in five independent fields (40×). The values are a mean number of percentage of scattered colonies ± SEM. The significance of the data was determined using Kruskal–Wallis test by ranks (^*^p ≤ 0.05, ^**^p ≤ 0.01, ^***^p ≤ 0.001, ^ns^*p* > 0.05). (**F**) Western blot analysis of whole cell lysates of Control and Plexin-B3 knockdown CD18/ HPAF cells. Quantification of phospho-MET(Tyr1234/1235) by the intensity of the bands with respect to the loading control GAPDH was performed by Image J software. Bands were normalized on the T3M-4 Control or shPlexin-B3 cells. (**G**) Western blot analysis showing no change in total cellular cMET receptor levels with knock down of Plexin-B3. Quantification of Met protein by the intensity of the bands with respect to the loading control GAPDH was performed using Image J software. Bands were normalized on the CD18/HPAF Control cells. (**H**) Bar graph showing inhibition of chemotaxis towards SEMA5A of T3M-4 cells with cMET inhibition. Transwell migration assay was utilized to evaluate chemotaxis of T3M-4- Control and Plexin-B3 knockdown cells towards SEMA5A in the presence and absence of 5 μg/mL cMET neutralization. The values are a mean number of percentage of migration ± SEM. The significance of the data was determined using Kruskal–Wallis test by ranks (^*^p ≤ 0.05, ^**^p ≤ 0.01, ^***^p ≤ 0.001, ^ns^*p* > 0.05.

Next, we generated stable Plexin-B3 knockdown in T3M-4 (Supplementary Figure 4A) and CD18/HPAF cell lines (Supplementary Figure 4B). We evaluated migration (Figure 5A), chemotaxis (Figure 5B) and scattering potential (Figure 5C–5E) of T3M4-Control and T3M-4-shPlexin-B3 cells treated with recombinant SEMA5A protein. We observed that significant inhibition in migration and chemotaxis in T3M-4-shPlexin-B3 knockdown cells treated with recombinant SEMA5A-protein compared to cells treated with recombinant SEMA5A protein. Scattering of colonies (Figure 5C) in both CD18/HPAF (Figure 5D) and T3M-4 (Figure 5E) Control cells were higher upon SEMA5A-treatment in comparison to their respective untreated control cells. On the other hand, both CD18/HPAF- and T3M-4-Plexin-B3 knockdown cells showed a lower scattering of colonies in response to SEMA5A treatment.

For evaluation of cMet activation on treatment with SEMA5A, we treated CD18/HPAF Plexin-B3 knockdown and CD18/HPAF Control cells with 50 ng of SEMA5A protein. We probed cell lysates of untreated- and SEMA5A treated-CD18/HPAF-shPlexin-B3 and CD18/HPAF-Control cells for phospho-cMet (Tyr1234/1235) and observed activation of cMet in CD18/HPAF-Control cells on treatment with 50 ng SEMA5A protein (Figure 5F). However, there was no activation of cMet receptor in CD18/HPAF-shPlexin-B3 cells upon SEMA5A treatment (Figure 5F). Total cellular cMet receptor levels remained unchanged with knock down of Plexin-B3 (Figure 5G). We also evaluated chemotaxis due to treatment of SEMA5A recombinant protein on T3M-4-Control and T3M-4-shPlexin-B3 cells in presence and absence of 5 μg cMet neutralization. We observed that cMet neutralization leads to a 60% decrease of chemotaxis in the T3M-4-Control cells upon SEMA5A treatment (Figure 5H). Whereas, knockdown of Plexin-B3 leads to a 30% decrease in chemotaxis upon SEMA5A treatment (Figure 5H). Additionally, knockdown of Plexin-B3 along with cMet neutralization resulted in 90% inhibition of chemotaxis even after treatment with SEMA5A (Figure 5H).

**Figure 6:**
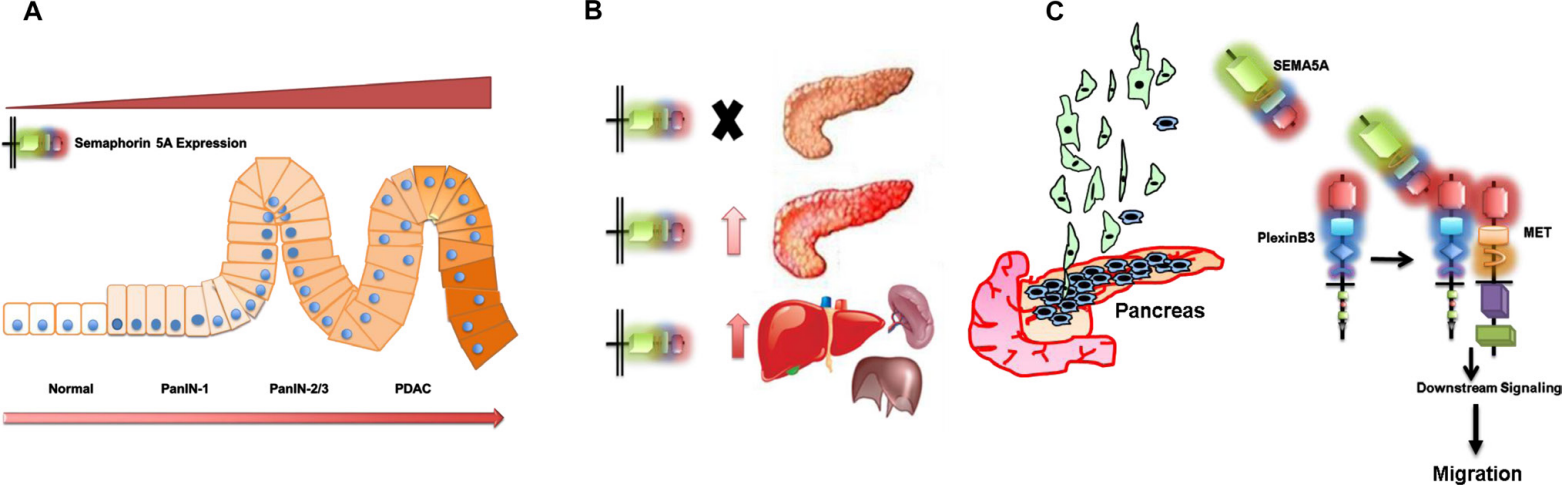
Schematic representation summarizing the role of SEMA5A in PC (**A**) SEMA5A expression analysis in different PC mouse models and human patient samples in a TMA suggests an increase in SEMA5A expression with the progression of PC with low or no expression in normal pancreas. (**B**) SEMA5A is also present in different PC metastatic sites like liver, spleen, and others. In pancreatic neuroendocrine cases, metastatic sites show furthermore upregulation of SEMA5A expression in comparison with the primary tumor. (**C**) The increase in SEMA5A during PC progression facilitates migration of PC cells. SEMA5A acts as a ligand and acts through Plexin-B3 receptor and activates cMET tyrosine kinase receptor for triggering PC cellular migration.

Together our data demonstrate that SEMA5A expression increases with the progression of PC with low or no expression in normal pancreas (Figure 6A) and different PC metastatic sites such as liver are also positive for SEMA5A expression (Figure 6B). SEMA5A as a ligand acts through Plexin-B3 receptor and activates cMET for triggering cellular migration (Figure 6C).

## DISCUSSION

In this present study, we determined the prospects of SEMA5A as a potential therapeutic target for PC by analyzing the pattern of SEMA5A expression in both human and murine PC tissues of the primary tumor as well as metastasis, and normal pancreas and its functional role in PC. We utilized human PC TMA of primary cancer and metastasis lesions as well as the human PanNET patient database. We also took advantage of KC and KPC mouse models for studying SEMA5A expression in PDAC and RT2 model of islet-cell carcinoma for PanNET. These mouse models of PC not only provide information about the progression of PC [29], but both KC and KPC models also bear a striking similarity to the PDAC condition [30] and KPC model also recapitulate histopathologic condition of metastasis to relevant sites. Our results demonstrated that an increased expression of SEMA5A in the metastatic lesions and primary tumor of human and mice PC tissue. The normal pancreas was either negative or showed low expression of SEMA5A. Localization of SEMA5A expression was mainly observed on the membrane of tumor cells with no staining for surrounding stroma. We compared SEMA5A expression in different metastatic lesions like liver, spleen, mesenchymal lymph node, peritoneum, and diaphragm in KPC mice and observed that positive SEMA5A expression in all above metastatic lesions except lymph node metatstatic lesions. Our observations are supported by a study of samples from gastric carcinoma patients illustrating elated expression levels of both SEMA5A and its receptor Plexin-B3 in primary gastric carcinoma and lymph node metastasis in comparison to the non-neoplastic tissue [16]. Since, recent literature indicates genomic aberrations in axonal guidance cue ligands, thus, in order to understand the cause of upregulation of SEMA5A in PC, we analyzed the gain of copy number variation in SEMA5A in PC patients. However, Our results does not reflect gain of copy number of SEMA5A as the cause of upregulation of SEMA5A in PC.

Next, we for the first time analyzed the expression of SEMA5A in the mouse PC progression using KC mice models with tumor sections obtained at different week/time points (10–50 week). We observed that progressive increase of of SEMA5A expression from pre-cancerous lesions (10-weeks of age) to pancreatic cancer (50 weeks of age). This observation does not go in hand with what we have seen for human PC patients. The possible reasons for this contrary results could be limited sample size of early staged PC patients as most of the diagnosis of PC occurs at an advanced stage. Another reason could be that our observations in KC mice progression model are at a qualitative level only. Lastly, in these mouse models, we are examining SEMA5A expression with Kras as a driver mutation, which may not reflect the entire representation of patient samples present in the TMA.

A mouse model different from the one described above was utilized to study the endocrine type of PC. The RT2 model is the most popular one for studying PanNET [31]. In this model, β-cell-specific expression of the SV40 T-antigen leads to islet-cell carcinomas [32]. The RT2 mouse model of islet cell carcinoma has been instrumental in studying PanNET progression and has been utilized for prediction of clinical efficacy of new therapeutics [33]. In endocrine tumors as well, we found a similar observation of highest *Sema5A* expression in liver metastases followed by primary tumors with no expression in the normal pancreas. Interestingly, in contrast to SEMA5A analysis in the human patient TMA, which revealed higher SEMA5A expression in differentiated tumors compared to poorly/undifferentiated tumors, in the RT2 model we observed downregulation of *Sema5A* expression in well-differentiated IT tumors, and higher *Sema5A* expression in poorly differentiated MLP. The reason for this contradictory observation could be a difference of analysis, examining protein levels using IHC versus examining RNA levels using microarray; also, though PDAC and PanNET represent malignancies of the pancreas, they have different pathophysiology. Additionally, Li *et al.* (2012) observed similar findings on SEMA5A in glioblastoma as we observed in regards to differentiation in human PC patient samples demonstrating that the treatment of glioblastoma cell lines with SEMA5A impaired cellular motility and promoted differentiation [11, 34].

We observed that *Sema5A* expression was high in angiogenic islets in the RT2 model which further supports our previous observations that SEMA5A can enhance angiogenesis [18]. However, we did not observe correlation between angiogenesis marker CD31 and SEMA5A in human pancreas and metastatic tissues, but a weak but significant correlation was present between SEMA5A and CXCL8 expression. Another interesting observation in human PC patients was the downregulation of *SEMA5A* expression in lymph node metastasis. In KPC mice, we also observe a similar loss of SEMA5A expression in lymph node metastatic lesions. Possible reasons for these observations can be that lymph node metastasis may represent cancer cells in transition and does not upregulate cell adhesion molecules such as SEMA5A.

In this study, we have also investigated the functional aspect of SEMA5A on PC cells and observed that the treatment of PC cells with SEMA5A acting as a ligand leads to an increase in cellular motility, induction of scattering as well as stimulation of a chemotactic effect in PC cells. Our present results further explain our previous findings that overexpression of secreted or membrane bound forms of SEMA5A enhances PC metastasis [19, 22]. Likewise, Pan *et al.* revealed that gastric cancer cells expressing SEMA5A have significantly higher invasion and metastasis potential through PI3K/Akt/uPA signal transduction pathway [14–16] but does not define whether SEMA5A increases invasion and metastasis acting as a ligand (forward signaling) or acting as a receptor (reverse manner). However, in contrast to our findings, SEMA5A acting as a ligand through Plexin-B3 receptor impedes cellular motility in glioblastoma patients [11, 12].

With the gain of knowledge that forward signaling of SEMA5A acting as a ligand induces a migratory phenotype in PC, we analyzed whether SEMA5A acts on PC cells using Plexin-B3 as the interacting receptor. Our *in vitro* studies suggest that SEMA5A induces migration in PC cells in a Plexin-B3 dependent manner. However, there is a need for future experiments to gain insight into whether SEMA5A acts only through Plexin-B3 in PC cells or there are other receptors which can mediate the effects of SEMA5A.

Plexins share high similarity with cMET and Ron members of the Scattered Factor Receptor family [35] and recently Conrotto *et al.* (2004). showed an association of cMET in receptor complexes formed by Plexin-B2 and Plexin-B3 but not with other family members [36]. In our experiments, we observed activation of cMet mediated in a Plexin-B3 manner. The chemotaxis of PC towards SEMA5A was inhibited by more than 50% by neutralization of cMET suggesting cMET activation as a necessary downstream event in a SEMA5A-mediated migratory response. Since we were able to inhibit only around 50% of the SEMA5A response, we cannot rule out the possibility of SEMA5A engaging other kinase receptors for chemotactic activity. Another possibility is that Plexin-B3 can elicit two different signaling pathways, one dependent on the cytoplasmic domain of Plexins and other dependent on engaging tyrosine kinase receptors such as cMET for mediating cellular migration. Also, other Semaphorins like Semaphorin-4D are known to engage cMET receptor using Plexin-B1 as a receptor and can initiate an invasive growth program [37] and angiogenesis [38].

Together, our data demonstrated that expression of SEMA5A in PC might be of diagnostic value for the occurrence and progression of PC and metastasis to distant organs (Figure 6). Our results also provide novel insights into the role of SEMA5A acting as a ligand in a forward manner on PC cells through Plexin-B3 receptor. Moreover, our results suggest the possible involvement of SEMA5A/Plexin-B3 with cMET receptor thereby inducing a migratory response in PC cells. Thus, the SEMA5A axis represents a potential target for the diagnosis and treatment of pancreatic tumors in the future.

## MATERIALS AND METHODS

### Human PC specimens

TMA slides were obtained from different sources. TMA obtained from the University of Nebraska Medical Center (UNMC) Rapid Autopsy Program were constructed from paraffin blocks containing 21 PC tumor cases, 3 non-matched normal pancreases, and 32 tissue cores from different metastatic sites including liver, diaphragm, and others. The second TMA set (Accumax Array, A207III) contained 33 cases and eight unmatched normal pancreatic tissues in duplicate (Petagene, Seoul, South Korea). The study was approved by the Institutional Review Board of the University of Nebraska Medical Center.

### Mouse model of PC disease progression specimens

Tissue section of different time points/ages (10–50 weeks) of primary tumor of the KC mouse model and different metastatic sites of KPC mice models were used.

### Cell lines

L3.3 and L3.6pl were obtained as a generous gift from Dr. I. J. Fidler of the University of Texas M.D. Anderson Cancer Center. T3M-4 and CD18/HPAF were obtained as generous gifts from Dr. Michael A. Hollingsworth and Dr. Surinder K. Batra, UNMC, Omaha NE respectively. These cell lines were maintained in culture as an adherent monolayer in Dulbecco’s Modified Eagle Medium (DMEM) (Hyclone Laboratories, Logan, UT, USA) supplemented with 5% fetal bovine serum (Sigma-Aldrich, St. Louis, MO, USA), 1% vitamin solution (Mediatech, Herndon, VA, USA), 1% L-glutamine (Mediatech) and 0.02% gentamycin (Invitrogen, Carlsbad, CA, USA). AsPC-1 was obtained from American Type Culture Collection (Manassas, VA, USA) and was cultured using RPMI-1640 (Sigma-Aldrich) supplemented with 5% fetal bovine serum, 1% L-glutamine and 0.02% gentamycin.

### Generation of Plexin-B3 knock-down cell lines

The pSuper.neo vector containing Plexin-B3 specific- or scrambled-oligo, their design, and sequence are previously described [18]. T3M-4 and CD18/HPAF were transfected with 1 µg pSuper-shPlexin-B3 or pSuper-scramble vector (Control) using Lipofectamine Plus reagent (Invitrogen). Control and Plexin-B3 knockdown T3M-4 and CD18/HPAF cell lines were selected with 1000 µg/mL or 600 µg/mL G418 Disulphate salt (Sigma-Aldrich) containing media respectively.

## IHC

IHC was performed as described previously [22]. IHC scoring was performed according to the following criteria: percentage of positive cells on the slides was as follows: 0 (negative), 0.1 (1%–10% of cells positive), 0.2 (11%–20% of cells positive), and 0.3 (20%-30% of cells positive) and so forth. Furthermore, the intensity was designated as weak (1 point), moderate (2 points), strong (3 points) or very strong (4). The IHC composite score was calculated by multiplying the extent of positive cells with intensity. Two independent observers examined each slide, and their observations were positively correlated with each other. Average scores were used for analysis and if the two observers significantly differed in their scoring, a third observer examined the slide.

### Immunoblotting

Western blotting was performed as described previously [18]. Briefly, the membrane was incubated with primary antibody against SEMA5A (Invitrogen; 1:1000), Plexin-B3 (Santa Cruz Biotechnology, Santa Cruz, CA; 1:200), cMET (Santa Cruz Biotechnology; 1:200) p-cMET (Cell Signaling Technologies, Danvers, MA, USA; 1:1000) overnight at 4°C The membrane was washed with TBST. After washing, the blots were incubated with secondary horseradish peroxidase-conjugated antibody [mouse (Sigma; 1:5000), or rabbit (Thermo Scientific; 1:5000)] for an hour at room temperature. The blots were developed using the Luminata™ Forte substrate (Millipore) on Molecular Imager^®^ Gel Doc™ XR System (Bio-Rad, Hercules, CA, USA) using Bio-Rad Image Lab version 5.2.1. The intensity of the bands obtained from immunoblotting was measured using NIH ImageJ software. Peaks were quantified for a protein of interest and their respective loading control. Bands were normalized on the control cells used in the study.

### Immunofluorescence

1 × 10^5^ cells were plated on 22x22mm coverslips (Fisherbrand, Pittsburgh, PA, USA), placed in 6-well plates, and allowed to adhere overnight. The next day, after washing with PBS, the cells were fixed using 4% paraformaldehyde in PBS for 10 minutes. Cells were washed with PBS for three times. For permeabiization, cells were treated with 0.1% Triton-X for 10 minutes followed by incubation with the respective primary antibodies in immunofluorescence solution (PBS with 5% BSA and 0.2% Saponin) for two hours at room temperature. Cells were washed three times with PBS. Next, cells were incubated with secondary antibodies conjugated with either FITC (Jackson Immunoresearch, West Grove, PA, USA) or Cy3 (Jackson Immunoresearch) for one hour at room temperature followed by three PBS washes. Nuclei were counterstained with 4, six diamidino-2-phenylindole (DAPI) for 5 minutes at room temperature. Finally, slides were mounted with Vectashield^®^ mounting medium (Vector Laboratories, Burlingame, CA, USA). Immunofluorescence was observed using a Nikon fluorescent Microscope (Melville, NY, USA).

### *In vitro* cell proliferation assay

Cells at different densities ranging between 2000–6000 were seeded and allowed to adhere overnight in 96-well plates. Cells were washed with HBSS, and incubated with medium alone or medium containing specified concentrations of SEMA5A-Fc (Sino Biologicals, Beijing) protein for 48 hours. Cell viability was determined by MTT assay (3- (4, 5 diMethylthiazol-2-yl)-2, 5- dipehnylate-tetrazolium bromide, tetrazole) as previously described [39]. Plates were read at 570 nm using Bio-Tex-ELx-800 plate reader (Houston, TX, USA).

### Wound healing assay

For this assay 2 × 10^5^ cells were seeded per well of a 12-well plate. The following day, when the cells were 90–95% confluent a wound was generated using a 1 mL pipette tip. Cells were washed with HBSS and incubated in serum-free medium alone or in serum-free medium with different concentrations of SEMA5A for 24 hours. Cells were photographed at different time points (T = 0 hour and T = 24 hours) using an inverted microscope at a 40× magnification. NIH ImageJ software was used to measure the distance migrated, which was calculated using the formula: 100* [Initial wound width (T = 0 hour) – Final wound width (T = 24 hour)]/(T = 0 hours).

### Chemotaxis assay

Chemotaxis assay was set up using Transwell chambers (Corning Costar Corp., Cambridge, MA, USA) with polycarbonate membranes containing 8.0 µm pores. Cells (1 × 10^5^) were plated onto Transwell chambers in serum-free media. Media (serum-free) alone or media containing SEMA5A protein was placed in the bottom of a 6-well plate, and the set up was incubated at 37°C in 5% CO2 for 24 hours. After 24 hours, cells from the top of the Transwell chambers were removed using a cotton swab (residual cells). We stained the membrane using Hema 3 kit (Fisher Scientific Company L.L.C., Kalamazoo, MI, USA) as per the manufacturer’s instructions and cells were counted in five independent high-power fields (200×), using a Nikon microscope.

### Cell scatter assay

Cells were seeded at a low density of 2x10^2^ cells per well in a 6-well plate in 3 mL of DMEM supplemented with 5% FBS and allowed to grow as discrete colonies at 37°C in a humidified atmosphere of 5% CO_2_ for 72 hours. When the majority of colonies contained 20–30 cells, the medium was replaced by DMEM containing 5% FBS or different concentrations of recombinant SEMA5A protein. After 16 hours of SEMA5A treatment, the medium was removed, and the cells were washed with PBS. The cells were fixed with methanol at room temperature for 10 minutes. After removal of methanol, the dish was allowed to air dry and stained with Giemsa stain at room temperature for 15 minutes. The stain was removed, and the dish was washed several times with distilled H_2_O and allowed to air dry. The percentage of scattered colonies was measured from the total number of colonies counted. When half of the cells in the given colony had lost contact with their neighbors and exhibited a fibroblast-like phenotype, it was judged as a “scattered” colony.

### PanNET

Methods for microarray analysis and parameters for hierarchical clustering for the progressive stages of the disease in RT2 mice are described in the previous study [27]. Similarly, Non-negative matrix factorization (NMF) consensus clustering of human mRNA transcriptomes of PanNET patients is also described in the same study.

### Copy number analysis

Online Tool was utilized to derive Copy Number Analysis for *SEMA5A* gene in PC patients using The Cancer Genome Atlas (TCGA) database.

### Pancreatic ductal adenocarcinoma database

The publicly available Gene Expression Omnibus (GEO) dataset GDS4103 consisting of 39 pancreatic ductal adenocarcinoma patients with tumor and the respective matched normal control samples as described by [40] were utilized for comparison of SEMA5A gene expression.

### Statistical analysis

Statistical analysis was conducted using GraphPad Prism software (GraphPad Software, Inc., La Jolla, CA, USA). The significance was determined by Student’s *t*-test or nonparametric Mann–Whitney *U-*test. Comparisons between different groups were evaluated using Kruskal–Wallis nonparametric Analysis of Variance followed by Bonferroni adjustment for multiple comparisons. For correlation analysis, nonparametric bivariate correlation analysis (Kendall’s tau_b and Spearman’s rho) was used (two-tailed). For all statistical tests, a *p*-value of ≤ 0.05 was considered significant.

## SUPPLEMENTARY MATERIALS FIGURES



## Abbreviations

PC: Pancreatic cancer
SEMA5A: Human Semaphorin-5A
Sema5A: Mouse Semaphorin-5A
PanNET: Pancreatic Neuroendocrine Tumors
cMET: Hepatocyte growth factor receptor
