# Supplementary material for: Pathological and functional significance of Semaphorin-5A in pancreatic cancer progression and metastasis
Source: Oncotarget. 2017 Dec 23;9(5):5931–43. doi: 10.18632/oncotarget.23644 (PMC5814185; doi:10.18632/oncotarget.23644)
Supplement: Supplementary file 1 [file oncotarget-09-5931-s001.pdf]

Pathological and functional significance of Semaphorin-5A in pancreatic cancer progression and metastasis

SUPPLEMENTARY MATERIALS

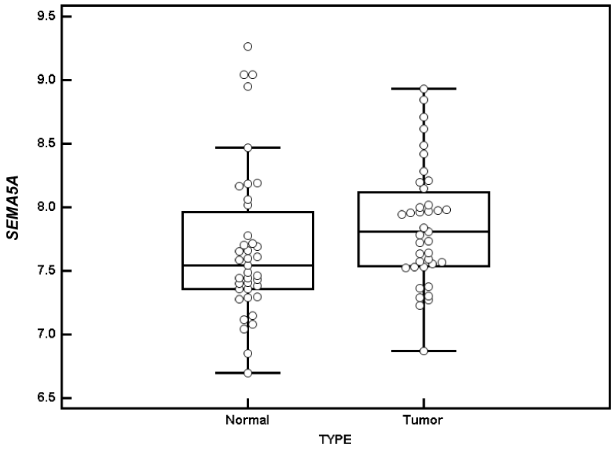

Supplementary Figure 1: Normal distribution of *SEMA5A* expression in matched tumor and normal samples. Box plot analysis showing normal distribution of *SEMA5A* expression values for both tumor samples and matched controls with four outliers present in the normal data. The data represents GEO PDAC microarray dataset GDS4103 containing values of 39 PDAC patients with matched tumor and normal samples.

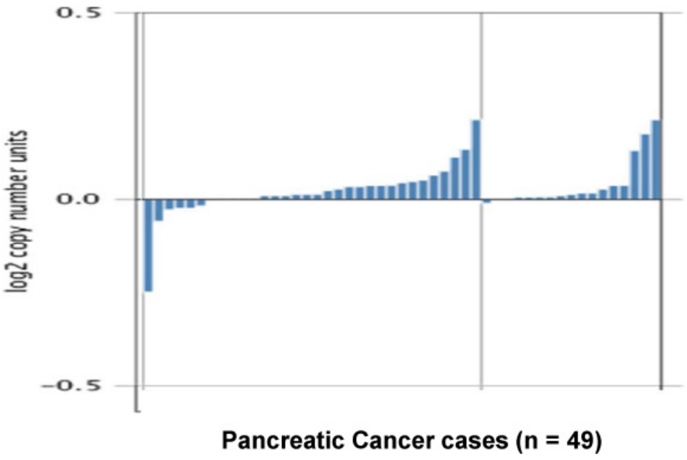

Supplementary Figure 2: Copy number analysis of *SEMA5A* in PC cases. Gain of *SEMA5A* gene copy number is observed in PC cases using The Cancer Genome Atlas (TCGA) Database. X axis represents log 2 value of copy number and Y axis represent individual pancreatic carcinoma cases ($n = 49$) cases. The gain of SEMA5A gene ranged between the value of 2 to 3 of copy number calculated by the formula $2*(\log_2 0-\log_2 0.5)$ in a diploid cell. However, aberrant gene expressions have a gain of copy number greater than 3.

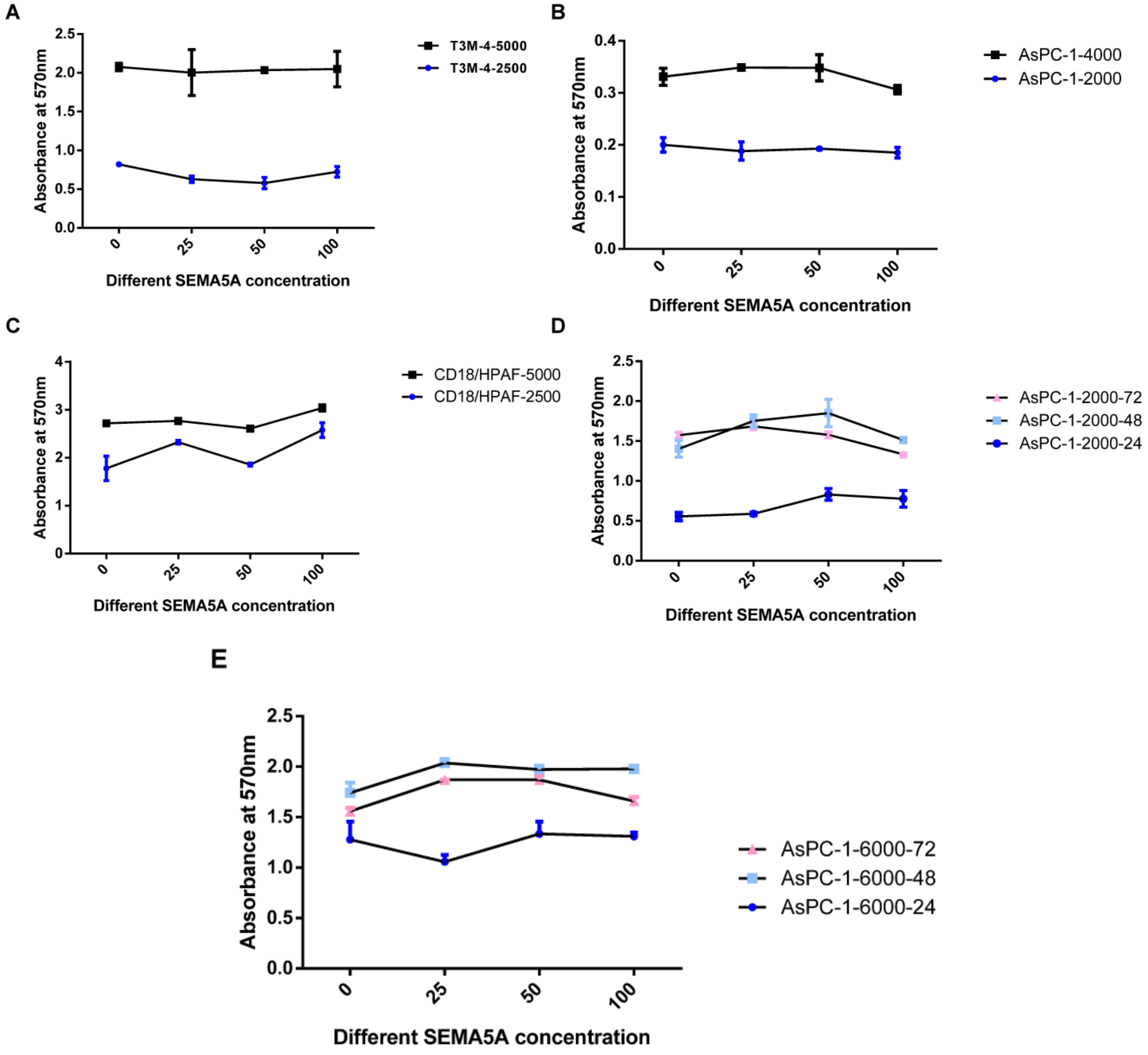

Supplementary Figure 3: Treatment with SEMA5A has no effect on cellular proliferation in PC cells. (**A**–**C**) Line graph demonstrating no effect of SEMA5A treatment at three different concentrations (25 ng, 50 ng, and 100 ng)/mL on cellular proliferation of AsPC-1 (B) T3M-4 (A) and CD18/HPAF (C) cells seeded at two different concentrations. Cellular proliferation was evaluated after 24 hours of SEMA5A treatment using the MTT assay. (**D**, **E**) Line graph demonstrating no effect of SEMA5A treatment at three different concentrations (25 ng, 50 ng, and 100 ng)/mL on proliferation of AsPC-1 seeded at 2000 cells per well (D) and at 6000 (E) cells per well and evaluated at different time points. The MTT assay was read using BIO-TEX ELx-800 plate reader at 570 nm wavelength. The values are mean of absorbance at 570 nm ± SEM. The significance of the data was calculated using Two-way ANOVA test.

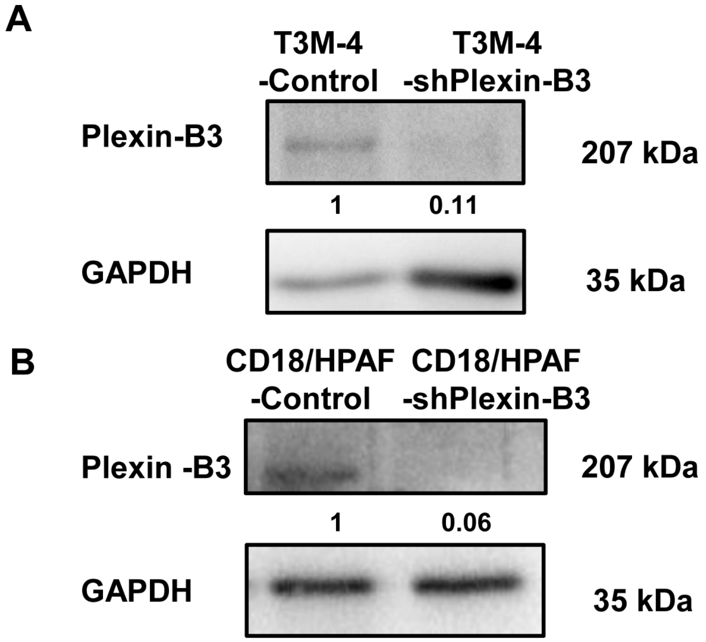

Supplementary Figure 4: Plexin-B3 expression in T3M-4 and CD18/HPAF Control and Plexin-B3 knockdown cells. Western blot analysis of whole cell lysates of T3M-4 and CD18/HPAF Control and Plexin-B3 knockdown cells. Plexin-B3 expression is downregulated in Plexin-B3 knockdown cells of T3M-4 and CD18/HPAF in comparison with their respective Control cells. Quantification of Plexin-B3 by the intensity of the bands with respect to the loading control GAPDH was performed using Image J software. Bands were normalized on the T3M-4 or CD18/HPAF control cells.
